# Supplementary material for: Habitat creation and biodiversity maintenance in mangrove forests: teredinid bivalves as ecosystem engineers
Source: PeerJ. 2014 Sep 25;2:e591. doi: 10.7717/peerj.591 (PMC4178455; doi:10.7717/peerj.591)
Supplement: Supplemental Information 1 — Total data from all LWD samples collected from each of the five transects within each mangrove forest locality. [file peerj-02-591-s001.docx]

| **Site** | **Area** | **SAMPLE** | **TRANSECT** | **SUBSTRATE** | **D.F.L** | **L.VOL** | **DECAY** | **ATTACK** | **sa cat** | **% SA** | **% SA arcsin** | **PSU** | **Sp per litre** | **ab per litre** |
| --- | --- | --- | --- | --- | --- | --- | --- | --- | --- | --- | --- | --- | --- | --- |
| Gili | 0.9 | GiW10.T1 | 1 | Mud | 93 | 0.38701464 | 0 | 0 | 0.-19.% | 0 | 0 | 35 | 0 | 0 |
| Gili | 0.9 | GiW8.T1 | 1 | Mud | 63 | 0.39091343 | 1 | 0 | 0.-19.% | 0 | 0 | 32 | 0 | 0 |
| Gili | 0.9 | GiW6.T1 | 1 | Mud | 30 | 0.43690325 | 2 | 0 | 0.-19.% | 0 | 0 | 35 | 0 | 0 |
| Gili | 0.9 | GiW4.T3 | 3 | Mud | 15 | 0.50588797 | 0 | 0 | 0.-19.% | 0 | 0 | 36 | 0 | 0 |
| Gili | 0.9 | GiW9.T3 | 3 | Mud | 35 | 0.80704965 | 1 | 0 | 0.-19.% | 0 | 0 | 35 | 0 | 0 |
| Gili | 0.9 | GiW10.T2 | 2 | Mud | 63 | 1.05410567 | 2 | 0 | 0.-19.% | 0 | 0 | 34 | 0 | 0 |
| Gili | 0.9 | GiW9.T2 | 2 | Mud | 54 | 1.53906747 | 1 | 0 | 0.-19.% | 0 | 0 | 34 | 0 | 0 |
| Gili | 0.9 | GiW8.T2 | 2 | Mud | 48 | 0.40738383 | 1 | 0 | 0.-19.% | 0 | 0 | 33 | 0 | 2.4546875 |
| Gili | 0.9 | GiW9.T1 | 1 | Mud | 66 | 0.32749841 | 1 | 0 | 0.-19.% | 0 | 0 | 32 | 3.053449951 | 3.053449951 |
| Gili | 0.9 | GiW8.T3 | 3 | Mud | 30 | 0.39091343 | 1 | 0 | 0.-19.% | 0 | 0 | 34 | 2.558111134 | 2.558111134 |
| Gili | 0.9 | GiW5.T3 | 3 | Mud | 18 | 0.59651496 | 0 | 0 | 0.-19.% | 0 | 0 | 35 | 1.676403895 | 1.676403895 |
| Gili | 0.9 | GiW1.T1 | 1 | Mud | 3 | 0.8042648 | 2 | 0 | 0.-19.% | 0 | 0 | 35 | 1.243371587 | 1.243371587 |
| Gili | 0.9 | GiW7.T3 | 3 | Mud | 24 | 0.66669319 | 0 | 1 | 0.-19.% | 0 | 0 | 35 | 1.499940327 | 4.499820981 |
| Gili | 0.9 | GiW5.T2 | 2 | Mud | 24 | 0.52633673 | 2 | 0 | 0.-19.% | 0 | 0 | 31 | 1.899924414 | 22.79909297 |
| Gili | 0.9 | GiW3.T2 | 2 | Mud | 15 | 0.11712285 | 3 | 1 | 0.-19.% | 2.23 | 8.588208008 | 33 | 8.538043478 | 8.538043478 |
| Gili | 0.9 | GiW4.T1 | 1 | Mud | 24 | 0.4898154 | 1 | 2 | 0.-19.% | 3.1 | 10.14082491 | 35 | 4.08317089 | 26.54061079 |
| Gili | 0.9 | GiW7.T2 | 2 | Mud | 42 | 0.4021324 | 0 | 1 | 0.-19.% | 3.75 | 11.1658225 | 36 | 0 | 0 |
| Gili | 0.9 | GiW2.T3 | 3 | Mud | 9 | 0.05155952 | 4 | 1 | 0.-19.% | 7.33 | 15.70828967 | 34 | 19.39506173 | 19.39506173 |
| Gili | 0.9 | GiW4.T2 | 2 | Mud | 18 | 0.75763845 | 1 | 1 | 0.-19.% | 9 | 17.45760312 | 31 | 1.319890779 | 2.639781558 |
| Gili | 0.9 | GiW6.T3 | 3 | Mud | 21 | 0.59293444 | 1 | 2 | 0.-19.% | 9.35 | 17.80495813 | 35 | 5.05958132 | 18.55179817 |
| Gili | 0.9 | GiW3.T1 | 1 | Mud | 21 | 0.36091661 | 3 | 1 | 0.-19.% | 10.95 | 19.32388707 | 35 | 8.312169312 | 8.312169312 |
| Gili | 0.9 | GiW3.T3 | 3 | Mud | 12 | 0.2989338 | 3 | 1 | 0.-19.% | 11.6 | 19.91268563 | 36 | 0 | 0 |
| Gili | 0.9 | GiW6.T2 | 2 | Mud | 30 | 0.7017823 | 2 | 2 | 0.-19.% | 18.96 | 25.81271086 | 33 | 8.549659864 | 9.974603175 |
| Gili | 0.9 | GiW1.T3 | 3 | Mud | 3 | 0.29630808 | 1 | 3 | 20.-39.% | 29.5 | 32.8975826 | 36 | 13.49946294 | 13.49946294 |
| Gili | 0.9 | GiW1.T2 | 2 | Mud | 3 | 0.56715468 | 2 | 3 | 20.-39.% | 34.6 | 36.03062877 | 34 | 7.052749719 | 14.10549944 |
| Gili | 0.9 | GiW7.T1 | 1 | Mud | 45 | 0.45989815 | 1 | 3 | 20.-39.% | 34.6 | 36.03062877 | 35 | 8.697577855 | 45.66228374 |
| Gili | 0.9 | GiW10.T3 | 3 | Mud | 35 | 0.39385742 | 1 | 2 | 40.-59.% | 40.29 | 39.40100319 | 35 | 5.077979798 | 12.69494949 |
| Gili | 0.9 | GiW2.T1 | 1 | Mud | 9 | 0.61616805 | 2 | 3 | 40.-59.% | 48.62 | 44.20921782 | 35 | 6.491735537 | 19.47520661 |
| Gili | 0.9 | GiW5.T1 | 1 | Mud | 27 | 0.4021324 | 2 | 3 | 40.-59.% | 49.24 | 44.56453531 | 35 | 7.460229521 | 29.84091808 |
| Gili | 0.9 | GiW2.T2 | 2 | Mud | 6 | 0.73201782 | 2 | 3 | > 60% | 75.73 | 60.48536109 | 34 | 6.830434783 | 8.196521739 |
| Kaluku | 1.2 | KaW2.T4 | 4 | Mud | 15 | 0.06462842 | 1 | 0 | 0.-19.% | 0 | 0 | 34 | 0 | 0 |
| Kaluku | 1.2 | KaW3.T6 | 6 | Mud | 30 | 0.06627944 | 1 | 0 | 0.-19.% | 0 | 0 | 35 | 0 | 0 |
| Kaluku | 1.2 | KaW1T2 | 2 | Mud | 6 | 1.19382559 | 2 | 0 | 0.-19.% | 0 | 0 | 31 | 0 | 0 |
| Kaluku | 1.2 | KaW3T2 | 2 | Mud | 15 | 0.32192871 | 2 | 1 | 0.-19.% | 7.11 | 15.4647714 | 32 | 3.106277805 | 3.106277805 |
| Kaluku | 1.2 | KaW4T5 | 5 | Mud | 27 | 0.23859563 | 1 | 1 | 0.-19.% | 8.96 | 17.41752141 | 28 | 4.191191687 | 8.382383374 |
| Kaluku | 1.2 | KaW1.T4 | 4 | Mud | 9 | 0.0377944 | 2 | 1 | 0.-19.% | 12.23 | 20.46983342 | 33 | 26.45894737 | 52.91789474 |
| Kaluku | 1.2 | KaW2.T6 | 6 | Mud | 24 | 0.11139402 | 2 | 1 | 0.-19.% | 13.3 | 21.38860759 | 33 | 17.95428571 | 62.84 |
| Kaluku | 1.2 | KaW1.T6 | 6 | Mud | 9 | 0.04575111 | 1 | 3 | 0.-19.% | 18.31 | 25.33448029 | 34 | 65.57217391 | 284.146087 |
| Kaluku | 1.2 | KaW2T3 | 3 | Mud | 6 | 0.61779575 | 0 | 2 | 20.-39.% | 24.33 | 29.5547157 | 31 | 0 | 0 |
| Kaluku | 1.2 | KaW5T5 | 5 | Mud | 33 | 0.712841 | 1 | 3 | 20.-39.% | 36.91 | 37.41155269 | 27 | 8.417024273 | 12.62553641 |
| Kaluku | 1.2 | KaW4T2 | 2 | Mud | 18 | 0.6354233 | 2 | 4 | 40.-59.% | 42.36 | 40.60538716 | 33 | 17.31129477 | 135.34285 |
| Kaluku | 1.2 | KaW1T5 | 5 | Mud | 9 | 2.3565 | 0 | 4 | 40.-59.% | 43.7 | 41.38074591 | 28 | 3.394865266 | 13.57946107 |
| Kaluku | 1.2 | KaW1T3 | 3 | Mud | 3 | 0.622116 | 0 | 3 | 40.-59.% | 45.22 | 42.25707277 | 31 | 3.214834532 | 4.822251799 |
| Kaluku | 1.2 | KaW3T5 | 5 | Mud | 21 | 0.678672 | 0 | 4 | 40.-59.% | 48.62 | 44.20921782 | 28 | 10.31426079 | 29.46931655 |
| Kaluku | 1.2 | KaW4.T6 | 6 | Mud | 42 | 0.06 | 1 | 2 | 40.-59.% | 53.65 | 47.09315784 | 36 | 33.33333333 | 100 |
| Kaluku | 1.2 | KaW3T3 | 3 | Mud | 18 | 1.02173913 | 0 | 4 | > 60% | 60.8 | 51.23710086 | 32 | 11.74468091 | 18.59574478 |
| Kaluku | 1.2 | KaW4T3 | 3 | Mud | 21 | 1.32553125 | 0 | 4 | > 60% | 61.37 | 51.57202352 | 33 | 8.29855954 | 109.390103 |
| Kaluku | 1.2 | KaW2T5 | 5 | Mud | 12 | 1.018008 | 0 | 4 | > 60% | 67.14 | 55.02377865 | 27 | 3.929242206 | 3.929242206 |
| Kaluku | 1.2 | KaW2T2 | 2 | Mud | 12 | 0.66669319 | 0 | 4 | > 60% | 69.89 | 56.72035885 | 33 | 7.499701635 | 10.49958229 |
| Langira | 9 | LanT5.D1 | 5 | Mud | 0 | 0.41797621 | 3 | 0 | 0.-19.% | 0 | 0 | 34 | 0 | 0 |
| Langira | 9 | LanT4.D5 | 4 | Roots | 80 | 0.63536362 | 2 | 0 | 0.-19.% | 0 | 0 | 34 | 0 | 0 |
| Langira | 9 | LanT5.D7 | 5 | Roots | 140 | 0.89906906 | 3 | 0 | 0.-19.% | 0 | 0 | 32 | 0 | 0 |
| Langira | 9 | LanT5.D4 | 5 | Mud | 80 | 0.57765754 | 0 | 0 | 0.-19.% | 0 | 0 | 34 | 1.731129477 | 1.731129477 |
| Langira | 9 | LanT3.D5 | 3 | Roots | 100 | 0.23150064 | 0 | 0 | 0.-19.% | 0 | 0 | 32 | 4.31964255 | 8.639285101 |
| Langira | 9 | LanT4.D3 | 4 | Mud | 50 | 0.54459739 | 4 | 0 | 0.-19.% | 0 | 0 | 33 | 1.836218862 | 3.672437724 |
| Langira | 9 | LanT4.D4 | 4 | Mud | 70 | 1.20377148 | 3 | 0 | 0.-19.% | 0 | 0 | 34 | 4.153612268 | 4.153612268 |
| Langira | 9 | LanT5.D3 | 5 | Mud | 70 | 0.38476687 | 0 | 0 | 0.-19.% | 0 | 0 | 33 | 2.598976374 | 15.59385824 |
| Langira | 9 | LanT4.D7 | 4 | Mud | 100 | 0.32224698 | 3 | 1 | 0.-19.% | 1.55 | 7.151824361 | 32 | 18.61925926 | 34.13530864 |
| Langira | 9 | LanT2:D5 | 2 | Sand/shell | 180 | 0.50063654 | 3 | 1 | 0.-19.% | 3.2 | 10.30484647 | 35 | 11.98474253 | 13.98219962 |
| Langira | 9 | LanT1.D6 | 1 | CORAL ROCK | 120 | 0.63136537 | 0 | 1 | 0.-19.% | 4.7 | 12.5208476 | 32 | 1.583868935 | 1.583868935 |
| Langira | 9 | LanT1.D8 | 1 | CORAL ROCK | 150 | 0.4898154 | 2 | 1 | 0.-19.% | 5.5 | 13.56337656 | 31 | 4.08317089 | 16.33268356 |
| Langira | 9 | LanT1.D2 | 1 | CORAL ROCK | 10 | 0.63136537 | 0 | 1 | 0.-19.% | 6.31 | 14.5483637 | 33 | 4.751606805 | 17.42255829 |
| Langira | 9 | LanT3.D1 | 3 | Sand/shell | 0 | 0.22915341 | 0 | 1 | 0.-19.% | 7 | 15.34170855 | 32 | 8.727777778 | 8.727777778 |
| Langira | 9 | LanT1.D13 | 1 | Sand/shell | 300 | 1.01758434 | 0 | 2 | 0.-19.% | 7.5 | 15.89416531 | 33 | 0 | 0 |
| Langira | 9 | LanT1.D10 | 1 | Sand/shell | 230 | 0.33438097 | 0 | 2 | 0.-19.% | 7.62 | 16.0242081 | 33 | 2.990600833 | 5.981201666 |
| Langira | 9 | LanT1.D12 | 1 | Roots | 280 | 0.38192234 | 0 | 2 | 0.-19.% | 8.04 | 16.47213112 | 34 | 2.618333333 | 2.618333333 |
| Langira | 9 | LanT2:D1 | 2 | CORAL ROCK | 100 | 0.54137492 | 0 | 1 | 0.-19.% | 8.11 | 16.54573548 | 36 | 1.847148736 | 1.847148736 |
| Langira | 9 | LanT5.D8 | 5 | CORAL ROCK | 160 | 1.00296388 | 0 | 1 | 0.-19.% | 8.13 | 16.56671204 | 32 | 0.997044882 | 2.991134646 |
| Langira | 9 | LanT1.D14 | 1 | Roots | 310 | 0.63136537 | 2 | 2 | 0.-19.% | 8.76 | 17.21589018 | 33 | 7.919344675 | 11.08708255 |
| Langira | 9 | LanT5.D2 | 5 | Mud | 40 | 0.58879694 | 3 | 1 | 0.-19.% | 8.88 | 17.33711518 | 33 | 0 | 0 |
| Langira | 9 | LanT5.D12 | 5 | Mud | 230 | 1.27880331 | 0 | 2 | 0.-19.% | 9.3 | 17.75569809 | 32 | 1.56396217 | 7.037829766 |
| Langira | 9 | LanT3.D3 | 3 | CORAL ROCK | 20 | 0.80149984 | 1 | 4 | 0.-19.% | 10.23 | 18.65347351 | 32 | 1.247660884 | 2.495321768 |
| Langira | 9 | LanT1.D1 | 1 | CORAL ROCK | 0 | 0.50588797 | 0 | 1 | 0.-19.% | 10.84 | 19.2227473 | 33 | 1.97672224 | 1.97672224 |
| Langira | 9 | LanT5.D6 | 5 | Mud | 110 | 1.10789306 | 3 | 2 | 0.-19.% | 11 | 19.3697123 | 32 | 0.902614191 | 1.805228383 |
| Langira | 9 | LanT4.D6 | 4 | Mud | 90 | 1.2063972 | 3 | 2 | 0.-19.% | 11.21 | 19.56119013 | 35 | 3.315657565 | 5.802400739 |
| Langira | 9 | LanT2:D2 | 2 | CORAL ROCK | 130 | 1.11394017 | 0 | 3 | 0.-19.% | 13.11 | 21.2278275 | 36 | 6.284 | 17.05657143 |
| Langira | 9 | LanT5.D13 | 5 | Roots | 250 | 0.5517286 | 0 | 2 | 0.-19.% | 14.7 | 22.54480537 | 32 | 3.624970706 | 5.43745606 |
| Langira | 9 | LanT3.D4 | 3 | CORAL ROCK | 80 | 0.850732 | 2 | 4 | 0.-19.% | 14.95 | 22.74635535 | 35 | 5.877291556 | 9.40366649 |
| Langira | 9 | LanT3.D6 | 3 | CORAL ROCK | 110 | 0.73559835 | 0 | 2 | 0.-19.% | 19.82 | 26.43591695 | 32 | 4.078312601 | 4.078312601 |
| Langira | 9 | LanT3.D11 | 3 | Sand/shell | 260 | 1.59137293 | 0 | 4 | 20.-39.% | 23.25 | 28.82804429 | 34 | 5.027105742 | 11.31098792 |
| Langira | 9 | LanT4.D13 | 4 | Mud | 220 | 0.54155395 | 0 | 4 | 20.-39.% | 23.29 | 28.85516318 | 35 | 5.539614325 | 11.07922865 |
| Langira | 9 | LanT3.D10 | 3 | Sand/shell | 250 | 0.68548098 | 0 | 4 | 20.-39.% | 24.1 | 29.40090459 | 32 | 1.458829675 | 2.917659349 |
| Langira | 9 | LanT4.D1 | 4 | Mud | 0 | 0.45383116 | 2 | 3 | 20.-39.% | 24.14 | 29.42769013 | 33 | 4.406925268 | 6.610387903 |
| Langira | 9 | LanT3.D8 | 3 | CORAL ROCK | 140 | 2.06452896 | 0 | 4 | 20.-39.% | 25.28 | 30.18490328 | 32 | 5.812463869 | 29.06231934 |
| Langira | 9 | LanT3.D7 | 3 | CORAL ROCK | 120 | 0.60510821 | 0 | 4 | 20.-39.% | 26.65 | 31.0801322 | 33 | 6.610387903 | 11.56817883 |
| Langira | 9 | LanT2:D3 | 2 | CORAL ROCK | 150 | 0.65181413 | 0 | 4 | 20.-39.% | 27.4 | 31.56396277 | 36 | 9.205078125 | 32.21777344 |
| Langira | 9 | LanT5.D16 | 5 | Sand/shell | 380 | 1.03226448 | 0 | 4 | 20.-39.% | 28.34 | 32.16459386 | 34 | 5.812463869 | 7.749951825 |
| Langira | 9 | LanT2:D9 | 2 | Sand/shell | 330 | 0.88391152 | 0 | 4 | 20.-39.% | 28.4 | 32.20272386 | 34 | 3.394004861 | 9.050679629 |
| Langira | 9 | LanT1.D11 | 1 | Sand/shell | 240 | 0.5549809 | 0 | 4 | 20.-39.% | 31.1 | 33.89507352 | 33 | 7.207455197 | 70.27268817 |
| Langira | 9 | LanT4.D8 | 4 | Mud | 120 | 0.36320417 | 0 | 4 | 20.-39.% | 31.35 | 34.04962277 | 32 | 13.76636179 | 30.28599595 |
| Langira | 9 | LanT4.D15 | 4 | Sand/shell | 290 | 0.64364855 | 0 | 4 | 20.-39.% | 31.4 | 34.08049228 | 35 | 6.214571583 | 9.321857375 |
| Langira | 9 | LanT2:D7 | 2 | Sand/shell | 230 | 1.34468491 | 0 | 4 | 20.-39.% | 32.26 | 34.60940633 | 35 | 7.436686391 | 33.46508876 |
| Langira | 9 | LanT4.D9 | 4 | CORAL ROCK | 150 | 0.90542449 | 0 | 4 | 20.-39.% | 33.49 | 35.35954244 | 33 | 4.417817323 | 11.04454331 |
| Langira | 9 | LanT2:D10 | 2 | Sand/shell | 350 | 0.46849141 | 1 | 3 | 20.-39.% | 33.77 | 35.52932856 | 34 | 2.13451087 | 2.13451087 |
| Langira | 9 | LanT5.D5 | 5 | Mud | 100 | 0.75302355 | 0 | 4 | 20.-39.% | 35.1 | 36.33124099 | 33 | 1.327979713 | 1.327979713 |
| Langira | 9 | LanT5.D15 | 5 | Sand/shell | 370 | 0.75095481 | 0 | 4 | 20.-39.% | 37.94 | 38.02131192 | 35 | 3.994914177 | 11.98474253 |
| Langira | 9 | LanT2:D11 | 2 | Sand/shell | 400 | 0.92425207 | 0 | 4 | 20.-39.% | 38.5 | 38.35146413 | 36 | 1.081955923 | 1.081955923 |
| Langira | 9 | LanT4.D16 | 4 | Sand/shell | 320 | 0.80681095 | 1 | 4 | 20.-39.% | 39 | 38.6454835 | 34 | 4.957790927 | 6.197238659 |
| Langira | 9 | LanT3.D2 | 3 | CORAL ROCK | 10 | 0.87141948 | 0 | 4 | 20.-39.% | 39.13 | 38.72181588 | 32 | 2.295105917 | 9.180423667 |
| Langira | 9 | LanT1.D4 | 1 | Roots | 50 | 1.99291852 | 0 | 4 | 20.-39.% | 39.32 | 38.83329733 | 30 | 0.50177666 | 0.50177666 |
| Langira | 9 | LanT1.D9 | 1 | Roots | 180 | 0.5360837 | 0 | 4 | 40.-59.% | 41.11 | 39.87917891 | 33 | 1.865380334 | 7.461521336 |
| Langira | 9 | LanT4.D2 | 4 | Mud | 20 | 0.22097788 | 0 | 4 | 40.-59.% | 41.2 | 39.93157141 | 34 | 0 | 0 |
| Langira | 9 | LanT5.D14 | 5 | Mud | 280 | 1.28952498 | 0 | 4 | 40.-59.% | 41.4 | 40.04793816 | 33 | 1.550958705 | 4.652876116 |
| Langira | 9 | LanT2.D4 | 2 | CORAL ROCK | 170 | 1.14377785 | 0 | 4 | 40.-59.% | 42.1 | 40.45458595 | 34 | 0 | 0 |
| Langira | 9 | LanT4.D14 | 4 | Sand/shell | 250 | 1.03481063 | 0 | 4 | 40.-59.% | 43.68 | 41.3691944 | 36 | 2.899081158 | 20.29356811 |
| Langira | 9 | LanT5.D9 | 5 | Mud | 170 | 0.5712325 | 0 | 4 | 40.-59.% | 45.7 | 42.53323438 | 32 | 3.501201379 | 5.251802068 |
| Langira | 9 | LanT2:D12 | 2 | Sand/shell | 440 | 0.52959898 | 0 | 3 | 40.-59.% | 49.13 | 44.50150156 | 36 | 5.664663462 | 5.664663462 |
| Langira | 9 | LanT4.D12 | 4 | Mud | 180 | 0.62633275 | 0 | 4 | 40.-59.% | 51.48 | 45.84810141 | 34 | 3.193190841 | 7.982977102 |
| Langira | 9 | LanT5.D10 | 5 | Sand/shell | 190 | 1.10264163 | 1 | 4 | 40.-59.% | 53.8 | 47.17934104 | 30 | 3.627651898 | 14.51060759 |
| Langira | 9 | LanT3.D12 | 3 | Sand/shell | 300 | 0.71809357 | 1 | 4 | 40.-59.% | 59.62 | 50.54643935 | 32 | 5.570304709 | 6.962880886 |
| Langira | 9 | LanT3.D9 | 3 | CORAL ROCK | 210 | 3.75167091 | 0 | 4 | > 60% | 63.23 | 52.67159305 | 32 | 2.932026892 | 11.19501177 |
| Langira | 9 | LanT1.D3 | 1 | CORAL ROCK | 30 | 0.49124761 | 2 | 4 | > 60% | 63.24 | 52.67753455 | 32 | 4.071266602 | 6.106899903 |
| Langira | 9 | LanT4.D11 | 4 | Mud | 170 | 0.98867163 | 0 | 4 | > 60% | 67.38 | 55.17029523 | 33 | 3.034374528 | 5.057290881 |
| Langira | 9 | LanT2.D6 | 2 | CORAL ROCK | 200 | 1.0101846 | 0 | 4 | > 60% | 68.47 | 55.83930925 | 34 | 5.939508507 | 10.88909893 |
| Langira | 9 | LanT1.D7 | 1 | CORAL ROCK | 140 | 0.47790022 | 0 | 4 | > 60% | 68.52 | 55.87014421 | 31 | 8.369947971 | 18.83238293 |
| Langira | 9 | LanT1.D5 | 1 | CORAL ROCK | 100 | 0.47175366 | 1 | 4 | > 60% | 73.16 | 58.79689561 | 31 | 19.07775342 | 69.95176252 |
| Langira | 9 | LanT4.D10 | 4 | Mud | 160 | 0.8042648 | 0 | 4 | > 60% | 74.82 | 59.88105518 | 33 | 4.973486347 | 12.43371587 |
| Langira | 9 | LanT5.D11 | 5 | CORAL ROCK | 220 | 0.8636219 | 0 | 4 | > 60% | 76.41 | 60.94195828 | 32 | 4.631656532 | 10.4212272 |
| Langira | 9 | LanT2. D8 | 2 | Sand/shell | 270 | 0.56476766 | 0 | 4 | > 60% | 86.4 | 68.359494 | 36 | 17.70639617 | 51.34854889 |
| Loho | 3.1 | LoW2T4 | 4 | Mud | 12 | 0.353475 | 0 | 0 | 0.-19.% | 0 | 0 | 35 | 0 | 0 |
| Loho | 3.1 | LoW4T4 | 4 | Mud | 78 | 0.876618 | 0 | 0 | 0.-19.% | 0 | 0 | 35 | 0 | 0 |
| Loho | 3.1 | LoW1T4 | 4 | Mud | 9 | 1.09302325 | 0 | 0 | 0.-19.% | 0 | 0 | 34 | 0 | 0 |
| Loho | 3.1 | LoW3.T5 | 5 | Mud | 36 | 3.054024 | 0 | 0 | 0.-19.% | 0 | 0 | 35 | 0 | 0 |
| Loho | 3.1 | LoW1T6 | 6 | Mud | 6 | 1.693538 | 1 | 1 | 0.-19.% | 2.99 | 9.957414519 | 33 | 0.590479812 | 0.590479812 |
| Loho | 3.1 | LoW5T5 | 5 | Mud | 54 | 1.80665 | 0 | 1 | 0.-19.% | 3.23 | 10.35356796 | 36 | 1.107021282 | 3.874574489 |
| Loho | 3.1 | LoW12T5 | 5 | Mud | 108 | 1.55980663 | 0 | 1 | 0.-19.% | 4.45 | 12.17804402 | 33 | 0 | 0 |
| Loho | 3.1 | LoW2.T5 | 5 | Mud | 33 | 1.53958 | 0 | 1 | 0.-19.% | 6.63 | 14.92105293 | 35 | 0 | 0 |
| Loho | 3.1 | LoW1T5 | 5 | Mud | 12 | 0.66276563 | 0 | 1 | 0.-19.% | 7.31 | 15.68629213 | 34 | 0 | 0 |
| Loho | 3.1 | LoW3T6 | 6 | Mud | 21 | 0.86405 | 0 | 2 | 0.-19.% | 8.86 | 17.31696262 | 35 | 4.629361727 | 4.629361727 |
| Loho | 3.1 | LoW10T5 | 5 | Mud | 90 | 1.29430763 | 0 | 1 | 0.-19.% | 9.23 | 17.6865339 | 34 | 1.545227704 | 3.090455409 |
| Loho | 3.1 | LoW3T4 | 4 | Mud | 73 | 0.54651163 | 0 | 2 | 0.-19.% | 9.87 | 18.31044686 | 35 | 5.489361731 | 10.97872346 |
| Loho | 3.1 | LoW9T5 | 5 | Mud | 84 | 1.654263 | 3 | 1 | 0.-19.% | 10.13 | 18.55873349 | 35 | 2.417995204 | 3.626992806 |
| Loho | 3.1 | LoW11T5 | 5 | Mud | 96 | 0.58215369 | 1 | 1 | 0.-19.% | 11.2 | 19.5521079 | 34 | 1.717759453 | 3.435518907 |
| Loho | 3.1 | LoW7T5 | 5 | Mud | 60 | 0.9426 | 3 | 1 | 0.-19.% | 11.43 | 19.76011269 | 35 | 2.121790791 | 3.182686187 |
| Loho | 3.1 | LoW3.T3 | 3 | Mud | 27 | 0.18292489 | 1 | 1 | 0.-19.% | 11.98 | 20.25026315 | 32 | 5.466724663 | 5.466724663 |
| Loho | 3.1 | LoW4.T1 | 1 | Mud | 21 | 0.32224698 | 1 | 2 | 0.-19.% | 19.23 | 26.00950462 | 32 | 3.103209877 | 18.61925926 |
| Loho | 3.1 | LoW7T6 | 6 | Mud | 54 | 0.64155713 | 0 | 3 | 20.-39.% | 25.02 | 30.01323013 | 35 | 3.117415304 | 4.676122956 |
| Loho | 3.1 | LoW5T4 | 4 | Mud | 105 | 1.2088845 | 0 | 4 | 20.-39.% | 25.84 | 30.55269576 | 35 | 1.654417771 | 25.64347545 |
| Loho | 3.1 | LoW3.T1 | 1 | Mud | 15 | 0.77196053 | 1 | 2 | 20.-39.% | 27.28 | 31.48683209 | 32 | 3.886209029 | 5.181612039 |
| Loho | 3.1 | LoW18T5 | 5 | Mud | 153 | 0.46334681 | 1 | 4 | 20.-39.% | 28.1 | 32.01182863 | 36 | 19.42389536 | 90.644845 |
| Loho | 3.1 | LoW2.T1 | 1 | Mud | 9 | 0.18332272 | 2 | 3 | 20.-39.% | 28.52 | 32.27891109 | 33 | 27.27430556 | 32.72916667 |
| Loho | 3.1 | LoW8T6 | 6 | Mud | 57 | 0.509004 | 0 | 4 | 20.-39.% | 29.14 | 32.67103122 | 34 | 9.823105516 | 13.75234772 |
| Loho | 3.1 | LoW19T5 | 5 | Mud | 165 | 0.63056013 | 2 | 4 | 20.-39.% | 30.02 | 33.22341253 | 36 | 11.10124114 | 34.88961501 |
| Loho | 3.1 | LoW2T6 | 6 | Mud | 12 | 0.552992 | 0 | 3 | 20.-39.% | 30.79 | 33.70295787 | 35 | 7.233377698 | 16.27509982 |
| Loho | 3.1 | LoW5.T1 | 1 | Mud | 24 | 0.2444303 | 0 | 4 | 20.-39.% | 31.09 | 33.8888845 | 32 | 28.63802083 | 53.18489583 |
| Loho | 3.1 | LoW4T5 | 5 | Mud | 39 | 1.385622 | 1 | 3 | 20.-39.% | 32.61 | 34.82359391 | 34 | 5.051882837 | 8.660370577 |
| Loho | 3.1 | LoW13T5 | 5 | Mud | 117 | 1.4633865 | 1 | 2 | 20.-39.% | 34.11 | 35.73503487 | 34 | 2.050039412 | 5.466771765 |
| Loho | 3.1 | LoW2.T3 | 3 | Mud | 6 | 0.29630808 | 1 | 2 | 20.-39.% | 36.37 | 37.09047962 | 32 | 10.12459721 | 23.62406015 |
| Loho | 3.1 | LoW4T6 | 6 | Mud | 30 | 0.760364 | 0 | 3 | 20.-39.% | 37.1 | 37.52428888 | 35 | 6.575797907 | 6.575797907 |
| Loho | 3.1 | LoW9T6 | 6 | Mud | 60 | 0.4909375 | 0 | 4 | 20.-39.% | 37.84 | 37.96225788 | 35 | 6.110757479 | 14.25843412 |
| Loho | 3.1 | LoW1.T3 | 3 | Mud | 3 | 0.04678549 | 1 | 2 | 40.-59.% | 40.76 | 39.67526391 | 32 | 64.12244898 | 64.12244898 |
| Loho | 3.1 | LoW1.T1 | 1 | Mud | 0 | 1.27140356 | 1 | 3 | 40.-59.% | 43.23 | 41.10912471 | 32 | 4.719193942 | 10.22492021 |
| Loho | 3.1 | LoW6T4 | 4 | Mud | 108 | 0.84834 | 0 | 4 | 40.-59.% | 43.55 | 41.29409501 | 25 | 15.3240446 | 44.79336115 |
| Loho | 3.1 | LoW16T5 | 5 | Mud | 141 | 1.52661925 | 1 | 3 | 40.-59.% | 45.6 | 42.47572052 | 35 | 2.620168716 | 14.41092794 |
| Loho | 3.1 | LoW17T5 | 5 | Mud | 147 | 0.593838 | 1 | 4 | 40.-59.% | 45.9 | 42.64823245 | 35 | 13.47168756 | 52.20278931 |
| Loho | 3.1 | LoW14T5 | 5 | Mud | 120 | 0.381753 | 1 | 3 | 40.-59.% | 46.65 | 43.07915244 | 34 | 7.858484413 | 13.09747402 |
| Loho | 3.1 | LoW5T6 | 6 | Mud | 33 | 2.2975875 | 0 | 4 | 40.-59.% | 47.37 | 43.49242527 | 35 | 2.176195684 | 3.046673957 |
| Loho | 3.1 | LoW8T5 | 5 | Mud | 66 | 2.281092 | 1 | 3 | 40.-59.% | 55.94 | 48.41142612 | 35 | 2.191932636 | 2.630319163 |
| Loho | 3.1 | LoW1.T2 | 2 | Mud | 6 | 0.42966264 | 0 | 4 | 40.-59.% | 57.5 | 49.31346328 | 30 | 16.29185185 | 114.042963 |
| Loho | 3.1 | LoW6T5 | 5 | Mud | 57 | 0.70695 | 1 | 4 | > 60% | 62.41 | 52.18551149 | 35 | 12.73074475 | 16.97432633 |
| Loho | 3.1 | LoW6T6 | 6 | Mud | 48 | 1.077706 | 0 | 4 | > 60% | 62.5 | 52.23875609 | 35 | 6.495277933 | 8.351071628 |
| Loho | 3.1 | LoW15T5 | 5 | Mud | 123 | 0.60444225 | 2 | 4 | > 60% | 70.8 | 57.29115352 | 34 | 9.926506627 | 23.1618488 |
| Loho | 3.1 | LoW4.T3 | 3 | Mud | 63 | 0.31826862 | 1 | 4 | > 60% | 78.96 | 62.69714728 | 32 | 28.278 | 62.84 |
| Onitu | 1 | OnW4.T5 | 5 | Sand | 36 | 0.02291534 | 1 | 0 | 0.-19.% | 0 | 0 | 33 | 0 | 0 |
| Onitu | 1 | OnW5.T5 | 5 | Sand | 72 | 0.04177276 | 1 | 0 | 0.-19.% | 0 | 0 | 36 | 0 | 0 |
| Onitu | 1 | OnW3.T5 | 5 | Sand | 27 | 0.09675366 | 1 | 0 | 0.-19.% | 0 | 0 | 32 | 0 | 0 |
| Onitu | 1 | OnW1.T3 | 3 | Sand | 9 | 0.10136856 | 0 | 0 | 0.-19.% | 0 | 0 | 32 | 0 | 0 |
| Onitu | 1 | OnW10T1 | 1 | Sand | 54 | 0.667675 | 0 | 0 | 0.-19.% | 0 | 0 | 35 | 0 | 0 |
| Onitu | 1 | OnW1T4 | 4 | Sand | 9 | 1.12837075 | 0 | 0 | 0.-19.% | 0 | 0 | 32 | 0 | 0 |
| Onitu | 1 | OnW1T2 | 2 | Sand | 3 | 1.13112 | 1 | 0 | 0.-19.% | 0 | 0 | 35 | 0 | 0 |
| Onitu | 1 | OnW3T1 | 1 | Sand | 9 | 1.13112 | 0 | 0 | 0.-19.% | 0 | 0 | 35 | 0 | 0 |
| Onitu | 1 | OnW9T2 | 2 | Sand | 87 | 1.05227562 | 1 | 0 | 0.-19.% | 0 | 0 | 35 | 0.950321361 | 0.950321361 |
| Onitu | 1 | OnW1.T5 | 5 | Sand | 9 | 0.04296626 | 1 | 0 | 0.-19.% | 0 | 0 | 32 | 46.54814815 | 46.54814815 |
| Onitu | 1 | OnW5T1 | 1 | Sand | 27 | 1.767375 | 1 | 0 | 0.-19.% | 0 | 0 | 35 | 1.131621755 | 1.697432633 |
| Onitu | 1 | OnW6T1 | 1 | Sand | 30 | 0.58853588 | 1 | 1 | 0.-19.% | 1.77 | 7.645375688 | 35 | 1.699131765 | 1.699131765 |
| Onitu | 1 | OnW3T4 | 4 | Sand | 18 | 0.6998805 | 2 | 3 | 0.-19.% | 3.89 | 11.37507198 | 32 | 1.428815348 | 1.428815348 |
| Onitu | 1 | OnW8T1 | 1 | Sand | 36 | 1.13112 | 0 | 1 | 0.-19.% | 3.98 | 11.50768529 | 34 | 1.768158993 | 4.420397482 |
| Onitu | 1 | OnW9T1 | 1 | Sand | 42 | 2.3541435 | 2 | 1 | 0.-19.% | 8.48 | 16.93007519 | 35 | 0.424782941 | 0.424782941 |
| Onitu | 1 | OnW5T2 | 2 | Sand | 21 | 0.791784 | 0 | 3 | 0.-19.% | 12.5 | 20.70481105 | 35 | 3.788912128 | 8.840794964 |
| Onitu | 1 | OnW6.T5 | 5 | Sand | 87 | 0.05458307 | 1 | 1 | 0.-19.% | 15.53 | 23.20868379 | 34 | 36.64139942 | 54.96209913 |
| Onitu | 1 | OnW1T1 | 1 | Sand | 3 | 0.364472 | 1 | 2 | 0.-19.% | 16.11 | 23.66401722 | 34 | 5.487389978 | 5.487389978 |
| Onitu | 1 | OnW2T2 | 2 | Sand | 6 | 0.87917088 | 3 | 3 | 0.-19.% | 19.51 | 26.21247847 | 35 | 2.274870627 | 2.274870627 |
| Onitu | 1 | OnW2T1 | 1 | Sand | 6 | 0.87722788 | 1 | 1 | 20.-39.% | 20.98 | 27.26065929 | 34 | 1.139954649 | 2.279909297 |
| Onitu | 1 | OnW8T2 | 2 | Sand | 66 | 0.56556 | 0 | 4 | 20.-39.% | 21.01 | 27.28176153 | 35 | 7.072635971 | 15.91343094 |
| Onitu | 1 | OnW7T1 | 1 | Sand | 33 | 0.6998805 | 0 | 4 | 20.-39.% | 32.1 | 34.51129006 | 33 | 7.144076739 | 11.43052278 |
| Onitu | 1 | OnW4T1 | 1 | Sand | 12 | 0.6873125 | 1 | 4 | 20.-39.% | 34.61 | 36.03665091 | 35 | 2.909884514 | 2.909884514 |
| Onitu | 1 | OnW3.T3 | 3 | Sand | 36 | 0.05728835 | 1 | 2 | 20.-39.% | 35.16 | 36.36724766 | 33 | 17.45555556 | 87.27777778 |
| Onitu | 1 | OnW2.T3 | 3 | Sand | 24 | 0.23074475 | 1 | 1 | 20.-39.% | 38.75 | 38.49856092 | 32 | 4.333793103 | 4.333793103 |
| Onitu | 1 | OnW2T4 | 4 | Sand | 15 | 1.12837075 | 0 | 4 | 40.-59.% | 45.64 | 42.49872728 | 32 | 2.658700609 | 7.976101826 |
| Onitu | 1 | OnW6T2 | 2 | Sand | 27 | 0.50272 | 1 | 4 | 40.-59.% | 47.49 | 43.56127123 | 35 | 1.989178867 | 1.989178867 |
| Onitu | 1 | OnW4.T3 | 3 | Sand | 99 | 0.16112349 | 1 | 2 | 40.-59.% | 51.35 | 45.77358703 | 35 | 6.206419753 | 31.03209877 |
| Onitu | 1 | OnW3T2 | 2 | Sand | 9 | 0.83164813 | 1 | 4 | 40.-59.% | 55.29 | 48.03662996 | 35 | 8.417021321 | 16.83404264 |
| Onitu | 1 | OnW7T2 | 2 | Sand | 30 | 0.6284 | 1 | 4 | > 60% | 60.46 | 51.03773717 | 35 | 3.182686187 | 3.182686187 |
| Onitu | 1 | OnW2.T5 | 5 | Sand | 12 | 0.06015277 | 1 | 2 | > 60% | 61.1 | 51.41326491 | 32 | 33.24867725 | 83.12169312 |
| Onitu | 1 | OnW4T2 | 2 | Sand | 18 | 1.9723905 | 0 | 4 | > 60% | 62.33 | 52.13820424 | 35 | 2.534994972 | 5.069989944 |
