# Supplementary material for: Habitat creation and biodiversity maintenance in mangrove forests: teredinid bivalves as ecosystem engineers
Source: PeerJ. 2014 Sep 25;2:e591. doi: 10.7717/peerj.591 (PMC4178455; doi:10.7717/peerj.591)
Supplement: Supplemental Information 2 — Raw data of LWD categorised by the percentage surface area of teredinid tunnels, with corresponding abundance of and numbers of species. [file peerj-02-591-s002.docx]

| **Site** | **SAMPLE** | **SA Cat** | **L. vol** | **% SA** | **Sp per litre** | **ab per litre** |
| --- | --- | --- | --- | --- | --- | --- |
| **Gili** | **GiW1.T2** | **20.-39.%** | **0.6** | **34.6** | **7.052749719** | **14.10549944** |
| **Gili** | **GiW10.T3** | **40.-59.%** | **0.4** | **40.29** | **5.077979798** | **12.69494949** |
| **Gili** | **GiW2.T2** | **> 60%** | **0.7** | **75.73** | **6.830434783** | **8.196521739** |
| **Gili** | **GiW7.T1** | **20.-39.%** | **0.5** | **34.6** | **8.697577855** | **45.66228374** |
| **Gili** | **GiW8.T2** | **0.-19.%** | **0.4** | **0** | **0** | **2.4546875** |
| **Gili** | **GiW8.T3** | **0.-19.%** | **0.4** | **0** | **2.558111134** | **2.558111134** |
| **Gili** | **GiW9.T1** | **0.-19.%** | **0.3** | **0** | **3.053449951** | **3.053449951** |
| **Kaluku** | **KaW1T3** | **40.-59.%** | **0.6** | **45.22** | **3.214834532** | **4.822251799** |
| **Kaluku** | **KaW1T5** | **40.-59.%** | **2.4** | **43.7** | **3.394865266** | **13.57946107** |
| **Kaluku** | **KaW2T2** | **> 60%** | **0.7** | **69.89** | **7.499701635** | **10.49958229** |
| **Kaluku** | **KaW2T5** | **> 60%** | **1** | **67.14** | **3.929242206** | **3.929242206** |
| **Kaluku** | **KaW3T2** | **0.-19.%** | **0.3** | **7.11** | **3.106277805** | **3.106277805** |
| **Kaluku** | **KaW3T3** | **> 60%** | **1** | **60.8** | **11.74468091** | **18.59574478** |
| **Kaluku** | **KaW3T5** | **40.-59.%** | **0.7** | **48.62** | **10.31426079** | **29.46931655** |
| **Kaluku** | **KaW4.T6** | **40.-59.%** | **0.1** | **53.65** | **33.33333333** | **100** |
| **Kaluku** | **KaW4T3** | **> 60%** | **1.3** | **61.37** | **8.29855954** | **109.390103** |
| **Kaluku** | **KaW5T5** | **20.-39.%** | **0.7** | **36.91** | **8.417024273** | **12.62553641** |
| **Langira** | **LanT1.D10** | **0.-19.%** | **0.3** | **7.62** | **2.990600833** | **5.981201666** |
| **Langira** | **LanT1.D12** | **0.-19.%** | **0.4** | **8.04** | **2.618333333** | **2.618333333** |
| **Langira** | **LanT1.D3** | **> 60%** | **0.5** | **63.24** | **4.071266602** | **6.106899903** |
| **Langira** | **LanT1.D5** | **> 60%** | **0.5** | **73.16** | **19.07775342** | **69.95176252** |
| **Langira** | **LanT1.D7** | **> 60%** | **0.5** | **68.52** | **8.369947971** | **18.83238293** |
| **Langira** | **LanT1.D9** | **40.-59.%** | **0.5** | **41.11** | **1.865380334** | **7.461521336** |
| **Langira** | **LanT2. D8** | **> 60%** | **0.6** | **86.4** | **17.70639617** | **51.34854889** |
| **Langira** | **LanT2.D6** | **> 60%** | **1** | **68.47** | **5.939508507** | **10.88909893** |
| **Langira** | **LanT2:D12** | **40.-59.%** | **0.5** | **49.13** | **5.664663462** | **5.664663462** |
| **Langira** | **LanT2:D3** | **20.-39.%** | **0.8** | **27.4** | **9.205078125** | **32.21777344** |
| **Langira** | **LanT2:D7** | **20.-39.%** | **1.3** | **32.26** | **7.436686391** | **33.46508876** |
| **Langira** | **LanT3.D11** | **20.-39.%** | **1.6** | **23.25** | **5.027105742** | **11.31098792** |
| **Langira** | **LanT3.D12** | **40.-59.%** | **0.7** | **59.62** | **5.570304709** | **6.962880886** |
| **Langira** | **LanT3.D7** | **20.-39.%** | **0.6** | **26.65** | **6.610387903** | **11.56817883** |
| **Langira** | **LanT3.D8** | **20.-39.%** | **2** | **25.28** | **5.812463869** | **29.06231934** |
| **Langira** | **LanT3.D9** | **> 60%** | **3.8** | **63.23** | **2.932026892** | **11.19501177** |
| **Langira** | **LanT4.D1** | **20.-39.%** | **0.5** | **24.14** | **4.406925268** | **6.610387903** |
| **Langira** | **LanT4.D10** | **> 60%** | **0.8** | **74.82** | **4.973486347** | **12.43371587** |
| **Langira** | **LanT4.D11** | **> 60%** | **1** | **67.38** | **3.034374528** | **5.057290881** |
| **Langira** | **LanT4.D12** | **40.-59.%** | **0.6** | **51.48** | **3.193190841** | **7.982977102** |
| **Langira** | **LanT4.D14** | **40.-59.%** | **1** | **43.68** | **2.899081158** | **20.29356811** |
| **Langira** | **LanT4.D15** | **20.-39.%** | **0.6** | **31.4** | **6.214571583** | **9.321857375** |
| **Langira** | **LanT4.D16** | **20.-39.%** | **0.8** | **39** | **4.957790927** | **6.197238659** |
| **Langira** | **LanT4.D6** | **0.-19.%** | **1.2** | **11.21** | **3.315657565** | **5.802400739** |
| **Langira** | **LanT4.D9** | **20.-39.%** | **0.9** | **33.49** | **4.417817323** | **11.04454331** |
| **Langira** | **LanT5.D10** | **40.-59.%** | **1** | **53.8** | **3.627651898** | **14.51060759** |
| **Langira** | **LanT5.D11** | **> 60%** | **0.9** | **76.41** | **4.631656532** | **10.4212272** |
| **Langira** | **LanT5.D15** | **20.-39.%** | **0.8** | **37.94** | **3.994914177** | **11.98474253** |
| **Langira** | **LanT5.D16** | **20.-39.%** | **1** | **28.34** | **5.812463869** | **7.749951825** |
| **Langira** | **LanT5.D6** | **0.-19.%** | **1** | **11** | **0.902614191** | **1.805228383** |
| **Langira** | **LanT5.D8** | **0.-19.%** | **1** | **8.13** | **0.997044882** | **2.991134646** |
| **Langira** | **LanT5.D9** | **40.-59.%** | **0.6** | **45.7** | **3.501201379** | **5.251802068** |
| **Loho** | **LoW1.T1** | **40.-59.%** | **1.3** | **43.23** | **4.719193942** | **10.22492021** |
| **Loho** | **LoW1.T3** | **40.-59.%** | **0.1** | **40.76** | **64.12244898** | **64.12244898** |
| **Loho** | **LoW15T5** | **> 60%** | **0.6** | **70.8** | **9.926506627** | **23.1618488** |
| **Loho** | **LoW16T5** | **40.-59.%** | **0.6** | **45.6** | **2.620168716** | **14.41092794** |
| **Loho** | **LoW17T5** | **40.-59.%** | **0.6** | **45.9** | **13.47168756** | **52.20278931** |
| **Loho** | **LoW1T6** | **0.-19.%** | **1.7** | **2.99** | **0.590479812** | **0.590479812** |
| **Loho** | **LoW2.T3** | **20.-39.%** | **0.3** | **36.37** | **10.12459721** | **23.62406015** |
| **Loho** | **LoW2T6** | **20.-39.%** | **0.6** | **30.79** | **7.233377698** | **16.27509982** |
| **Loho** | **LoW4.T3** | **> 60%** | **0.5** | **78.96** | **28.278** | **62.84** |
| **Loho** | **LoW4T5** | **20.-39.%** | **1.4** | **32.61** | **5.051882837** | **8.660370577** |
| **Loho** | **LoW5T5** | **0.-19.%** | **1.8** | **3.23** | **1.107021282** | **3.874574489** |
| **Loho** | **LoW6T4** | **40.-59.%** | **0.8** | **43.55** | **15.3240446** | **44.79336115** |
| **Loho** | **LoW6T5** | **> 60%** | **0.7** | **62.41** | **12.73074475** | **16.97432633** |
| **Loho** | **LoW6T6** | **> 60%** | **1** | **62.5** | **6.495277933** | **8.351071628** |
| **Loho** | **LoW7T5** | **0.-19.%** | **0.9** | **11.43** | **2.121790791** | **3.182686187** |
| **Loho** | **LoW8T6** | **20.-39.%** | **0.5** | **29.14** | **9.823105516** | **13.75234772** |
| **Loho** | **LoW9T5** | **0.-19.%** | **1.7** | **10.13** | **2.417995204** | **3.626992806** |
| **Loho** | **LoW9T6** | **20.-39.%** | **0.5** | **37.84** | **6.110757479** | **14.25843412** |
| **Onitu** | **OnW10T1** | **0.-19.%** | **0.7** | **0** | **0** | **0** |
| **Onitu** | **OnW1T2** | **0.-19.%** | **1** | **0** | **0** | **0** |
| **Onitu** | **OnW1T4** | **0.-19.%** | **1** | **0** | **0** | **0** |
| **Onitu** | **OnW2.T5** | **> 60%** | **0.1** | **61.1** | **33.24867725** | **83.12169312** |
| **Onitu** | **OnW2T2** | **0.-19.%** | **0.9** | **19.51** | **2.274870627** | **2.274870627** |
| **Onitu** | **OnW2T4** | **40.-59.%** | **1** | **45.64** | **2.658700609** | **7.976101826** |
| **Onitu** | **OnW3T1** | **0.-19.%** | **1** | **0** | **0** | **0** |
| **Onitu** | **OnW3T2** | **40.-59.%** | **0.8** | **55.29** | **8.417021321** | **16.83404264** |
| **Onitu** | **OnW4.T3** | **40.-59.%** | **0.2** | **51.35** | **6.206419753** | **31.03209877** |
| **Onitu** | **OnW7T1** | **20.-39.%** | **0.7** | **32.1** | **7.144076739** | **11.43052278** |
| **Onitu** | **OnW7T2** | **> 60%** | **0.6** | **60.46** | **3.182686187** | **3.182686187** |
| **Onitu** | **OnW9T1** | **0.-19.%** | **2.4** | **8.48** | **0.424782941** | **0.424782941** |
| **Onitu** | **OnW9T2** | **0.-19.%** | **1** | **0** | **0.950321361** | **0.950321361** |
