# Supplementary material for: Habitat creation and biodiversity maintenance in mangrove forests: teredinid bivalves as ecosystem engineers
Source: PeerJ. 2014 Sep 25;2:e591. doi: 10.7717/peerj.591 (PMC4178455; doi:10.7717/peerj.591)
Supplement: Supplemental Information 3 — Raw data of internal wood (n = 27) and outside air temperatures, and the abundance of animals removed from the same samples of wood. [file peerj-02-591-s003.docx]

| **DFL** | **Length** | **Circumference** | **°C_ambient_** | **°C_internal_** | **°C_difference_** | **Sample** | **Length** | **Circumference** | **Lv** | **Habitat** | **Attack** | **Image #** | **Abundance/ L** | **Species** |
| --- | --- | --- | --- | --- | --- | --- | --- | --- | --- | --- | --- | --- | --- | --- |
| **124** | **182** | **24** | **26.4** | **25.7** | **-0.7** | **W12.T1** | **13** | **19** | **0.373409** | **Covered** | **0** | **883** | **0** | **0** |
| **7** | **573** | **26** | **27** | **26** | **-1** | **W4.T1** | **16** | **17** | **0.367919** | **Covered** | **1** | **835** | **0** | **0** |
| **8.9** | **192** | **46** | **28.6** | **26.5** | **-2.1** | **W9.T1** | **12** | **17** | **0.275939** | **Covered** | **1** | **839** | **0** | **0** |
| **13** | **33** | **19** | **26.1** | **25** | **-1.1** | **W10.T1** | **23** | **21** | **0.80705** | **Covered** | **2** | **883** | **1** | **1** |
| **121** | **76** | **19** | **26.3** | **25.6** | **-0.7** | **W13.T1** | **18** | **27** | **1.04408** | **Covered** | **3** | **884** | **2** | **1** |
| **5** | **400** | **51** | **26.7** | **25** | **-1.7** | **W2.T1** | **12** | **28** | **0.748568** | **Covered** | **3** | **840** | **3** | **2** |
| **3** | **183** | **22** | **26** | **25.8** | **-0.2** | **W3.T1** | **17** | **25** | **0.845401** | **Covered** | **0** | **847** | **3** | **1** |
| **205** | **96** | **42** | **28.6** | **26.4** | **-2.2** | **W20.T1** | **19** | **28.5** | **1.22794** | **Covered** | **4** | **892** | **5** | **3** |
| **9.2** | **271** | **19** | **26.1** | **25.4** | **-0.7** | **W8.T1** | **16** | **25** | **0.795672** | **Covered** | **2** | **836** | **7** | **3** |
| **3** | **463** | **65** | **26.1** | **25** | **-1.1** | **W1.T1** | **20** | **27** | **1.160089** | **Covered** | **1** | **845** | **8** | **3** |
| **177** | **286** | **73** | **30.4** | **26** | **-4.4** | **W15.T1** | **20** | **21** | **0.701782** | **Exposed** | **1** | **893** | **0** | **0** |
| **203** | **112** | **73** | **28.7** | **26** | **-2.7** | **W19.T1** | **12.5** | **20** | **0.397836** | **Exposed** | **2** | **887** | **0** | **0** |
| **260** | **106** | **23** | **31.5** | **28** | **-3.5** | **W26.T1** | **14** | **22** | **0.539147** | **Exposed** | **1** | **880** | **0** | **0** |
| **9.8** | **210** | **33** | **31** | **26.9** | **-4.1** | **W6.T1** | **18** | **25.5** | **0.931294** | **Exposed** | **0** | **842** | **0** | **0** |
| **12** | **562** | **53** | **34.5** | **26.3** | **-8.2** | **W7.T1** | **16** | **22** | **0.616168** | **Exposed** | **2** | **841** | **0** | **0** |
| **102** | **173** | **21** | **29.2** | **24.9** | **-4.3** | **W11.T1** | **21** | **21** | **0.736871** | **Exposed** | **2** | **888** | **1** | **1** |
| **140** | **230** | **27** | **28.9** | **26.7** | **-2.2** | **W14.T1** | **21** | **28** | **1.309994** | **Exposed** | **3** | **878** | **1** | **1** |
| **6** | **187** | **36** | **27.5** | **26.4** | **-1.1** | **W5.T1** | **20** | **22** | **0.77021** | **Exposed** | **3** | **843** | **4** | **3** |
| **192** | **132** | **38** | **28.3** | **25.6** | **-2.7** | **W18.T1** | **17** | **25** | **0.845401** | **Exposed** | **2** | **891** | **5** | **2** |
| **242** | **323** | **23** | **32** | **29.4** | **-2.6** | **W23.T1** | **23** | **27** | **1.334102** | **Exposed** | **3** | **885** | **6** | **5** |
| **192** | **306** | **23** | **26.3** | **25.7** | **-0.6** | **W17.T1** | **21** | **27** | **1.218094** | **Exposed** | **3** | **894** | **8** | **5** |
| **242** | **103** | **32** | **30.8** | **27.7** | **-3.1** | **W25.T1** | **16** | **25** | **0.795672** | **Exposed** | **3** | **881** | **8** | **5** |
| **222** | **189** | **21** | **28** | **27.6** | **-0.4** | **W22.T1** | **21** | **27** | **1.218094** | **Exposed** | **4** | **890** | **11** | **3** |
| **307** | **507** | **24** | **30.5** | **27.6** | **-2.9** | **W27.T1** | **19** | **22** | **0.7317** | **Exposed** | **3** | **879** | **11** | **6** |
| **242** | **117** | **41** | **28.9** | **27.1** | **-1.8** | **W24.T1** | **21** | **23** | **0.883912** | **Exposed** | **3** | **877** | **12** | **6** |
| **222** | **207** | **47** | **29.6** | **25** | **-4.6** | **W21.T1** | **20** | **23** | **0.84182** | **Exposed** | **4** | **889** | **16** | **5** |
| **182** | **736** | **56** | **35.8** | **26.3** | **-9.5** | **W16.T1** | **24** | **37** | **2.614258** | **Exposed** | **4** | **868 + 895** | **42** | **8** |
